# Plasmid-Encoded Traits Vary across Environments

**DOI:** 10.1128/mbio.03191-22

**Published:** 2023-01-11

**Authors:** Sarai S. Finks, Jennifer B. H. Martiny

**Affiliations:** a Department of Ecology and Evolutionary Biology, University of California—Irvine, Irvine, California, USA; Georgia Institute of Technology

**Keywords:** accessory traits, horizontal gene transfer, HGT, meta-analysis, plasmids, replicon types

## Abstract

Plasmids are key mobile genetic elements in bacterial evolution and ecology as they allow the rapid adaptation of bacteria under selective environmental changes. However, the genetic information associated with plasmids is usually considered separately from information about their environmental origin. To broadly understand what kinds of traits may become mobilized by plasmids in different environments, we analyzed the properties and accessory traits of 9,725 unique plasmid sequences from a publicly available database with known bacterial hosts and isolation sources. Although most plasmid research focuses on resistance traits, such genes made up <1% of the total genetic information carried by plasmids. Similar to traits encoded on the bacterial chromosome, plasmid accessory trait compositions (including general Clusters of Orthologous Genes [COG] functions, resistance genes, and carbon and nitrogen genes) varied across seven broadly defined environment types (human, animal, wastewater, plant, soil, marine, and freshwater). Despite their potential for horizontal gene transfer, plasmid traits strongly varied with their host’s taxonomic assignment. However, the trait differences across environments of broad COG categories could not be entirely explained by plasmid host taxonomy, suggesting that environmental selection acts on the plasmid traits themselves. Finally, some plasmid traits and environments (e.g., resistance genes in human-related environments) were more often associated with mobilizable plasmids (those having at least one detected relaxase) than others. Overall, these findings underscore the high level of diversity of traits encoded by plasmids and provide a baseline to investigate the potential of plasmids to serve as reservoirs of adaptive traits for microbial communities.

## INTRODUCTION

The acquisition of new traits from mobile genetic elements such as plasmids is broadly thought to be important for bacterial diversification and adaptation (1, 2). Plasmids carry genes that encode a diversity of traits involved in plasmid-specific functions as well as those related to the physiology of their host (3–6) and may offer “snapshots” of events of horizontal gene transfer (HGT) between bacteria and plasmids spanning recent and historical timescales. However, analyses of bacterial traits and their corresponding ecological roles typically focus on chromosomal genetic content (7). Furthermore, in nonclinical microbial genomics studies, plasmids are often not distinguished from chromosomal sequences or are inadvertently removed, potentially obscuring our understanding of trait variation in environmental communities (8). Indeed, understanding what kinds of traits are carried by plasmids, and why, remains an open question in plasmid ecology (8, 9). Some evidence suggests that ecology (or the local biotic and abiotic environment), rather than geographic isolation and host phylogeny, drives plasmid-mediated gene exchange in bacteria (10–12). To begin to understand the extent to which some traits may be limited by ecological opportunity and the occupancy of shared habitats, the first step is to characterize the accessory trait variation on plasmids across different environments.

Many studies have focused on the intrinsic properties of plasmids, including their structure (linear or circular forms), size, copy number, mechanisms of replication (incompatibility and replicon types) and segregation, GC content, host range, and mobility (13–16), but this information is usually considered separately from their environmental origin. Indeed, much of our understanding of the diversity and ecological significance of plasmid properties is derived from a limited number of bacterial taxa and environments, particularly those within the phyla *Proteobacteria* and *Firmicutes* that were isolated from human and other host-associated environments. Finally, these studies generally do not address the full diversity of accessory traits (17) encoded by plasmids, traits that may contribute to host fitness.

Perhaps the best-studied plasmid accessory traits are those for antibiotic resistance and virulence (17–20). For instance, plasmids carrying and transferring genes encoding extended-spectrum β-lactamases are responsible for multidrug resistance in pathogenic bacterial hosts (21–24). Plasmid virulence genes, such as those encoding fimbriae (important for bacterial attachment to the human reticuloendothelial system), underlie the ability of bacterial pathogens to establish infections (25, 26). More broadly, resistance genes, involved in resistance to heavy metals, biocides, and antibiotics, are commonly reported from plasmids in human-impacted environments. For example, plasmids from wastewater treatment plants are considered reservoirs of resistance traits (27). Similarly, plasmids from environments contaminated with petroleum and other pollutants encode resistance to antibiotics, heavy metals, and herbicides as well as pathways to degrade xenobiotics and protect against UV radiation and exogenous DNA (28–36).

Even in less-heavily human-impacted environments, plasmid accessory traits reveal a connection between plasmid genetic contents and their ecological roles. The plasmidome (i.e., plasmid content assessed by culture-independent methods) in the bovine rumen includes genes enriched for amino acid, cell wall and capsule, vitamin, and protein metabolism functions, suggesting their importance in conferring nutritional advantages to their bacterial hosts (37). In rhizosphere bacteria, plasmid genes for nitrogen fixation aid in establishing symbiotic relationships between plant and host bacteria (38–40). Plasmids from aquatic environments are generally less well studied (38), but those from marine *Lentimonas* species encode putative carbohydrate-active enzymes (CAZymes), including fucoidanases, glycoside hydrolases (GHs), sulfatases, and carbohydrate esterases, which are important for degrading recalcitrant polysaccharides (41). Yet despite these individual examples, it remains unclear how plasmid diversity contributes to the trait diversity of bacterial communities.

To begin to address this knowledge gap, we analyzed a publicly available database (PLSDB) containing over 23,000 plasmid sequences collected from the NCBI (42). To investigate the potential link between plasmid traits and host bacterial ecology, we first asked, do plasmid properties and accessory genes vary by environment? We investigated genes encoding mobility and replication (i.e., replicon types/incompatibility groups). In contrast, we then characterized plasmid accessory genes by assigning Clusters of Orthologous Genes (COG) functions to all gene calls and focusing on three specific gene functions of interest, namely, resistance genes, carbon utilization genes (CAZymes), and genes encoding inorganic/organic nitrogen processing. While the latter two functions are not often considered plasmid associated, they are central to biogeochemical processes and ecosystem functioning. We hypothesized that plasmid accessory traits are subject to similar selection pressures experienced by the host microbiomes for these traits (43, 44) and would therefore vary by environment.

Second, we asked, to what extent does host taxonomy influence plasmid traits across environments? Both the environment and host phylogeny likely shape the accessory genes on a plasmid. Indeed, plasmid genetic similarity appears to decrease with phylogenetic distance, especially at taxonomic ranks broader than order (45). Moreover, the composition of bacterial communities varies dramatically across environments, making it difficult to fully separate the influence of the environment from that of the host composition. To disentangle these factors, we tested the effect of the environment versus the host bacterial phylum (as a broad metric of phylogenetic distinctiveness) on the plasmid accessory gene composition. Given the ability of many plasmids to be horizontally transferred and, thereby, break up a phylogenetic signal of the host, we hypothesized that plasmid traits would vary more strongly with environment than with bacterial host taxonomy (46–48). Moreover, we expected that genes involved in plasmid mobility (those encoding a relaxase), a trait intrinsic to plasmids, would be more closely associated with host taxonomy than accessory traits, which may be directly selected by the host’s environment (46, 47).

Third, we asked, are certain accessory traits and/or environments more often associated with mobilizable plasmids? We hypothesized that some accessory traits, those that might confer a sudden and strong selective advantage, such as resistance traits, would be more frequently associated with mobilizable plasmids. Here, we define mobilizable plasmids as those that carry genes necessary to hitchhike with self-transmissible/conjugative plasmids during transfer. Similarly, we expected that mobilizable plasmids would therefore be more abundant in environments that are reservoirs of these traits.

Finally, we note that a limitation of the PLSDB is that it contains plasmids derived from mainly cultured strains and, thus, represents a biased sampling of plasmids in an environment. Thus, we also consider the sensitivity of the observed patterns to the taxonomic biases of the database. We note, however, that this database has two large advantages compared to most culture-independent methods that allow us to address our questions: we have the full sequence of each plasmid, and the host bacterium of each plasmid included in the data set analyzed here is known.

## RESULTS

After filtering the PLSDB by our criteria, we analyzed 9,725 unique plasmid sequences from human, animal, wastewater, plant, soil, marine, and freshwater environments. As expected, the taxonomic representation of the plasmid sequences was highly skewed (see Fig. S1 in the supplemental material) and varied dramatically across environments (Fig. S2). The bulk of the plasmid sequences were associated with three bacterial host phyla, namely, *Proteobacteria* (*n* = 7,319), *Firmicutes* (*n* = 1,565), and *Actinobacteria* (*n* = 304). Sampling was also uneven; the majority (52.6%) of the plasmid sequences were from human environments, whereas 11.2% and 2.2% of the plasmids were from plant and marine environments, respectively (Table 1).

**TABLE 1:** Summary of plasmid genetic contents across environments

Parameter	Value for environment							
	Human	Animal	Wastewater	Plant	Soil	Marine	Freshwater	Total
No. of plasmid sequences	5,122	2,316	227	1,089	657	213	101	9,725
No. of gene calls^*a*^	439,164	201,694	21,017	422,909	136,619	27,715	14,567	1,263,685
Mean plasmid size (kb)	76.3	79.3	88.4	426.9	215.5	130.1	146.6	
No. of MOBs	2,686	1,077	154	633	325	96	54	5,025
% mobilizable plasmids^*b*^	50.5	43.7	59.5	52.5	45.7	41.3	47.5	
No. of assigned COGs	227,768	104,664	11,742	319,138	89,239	17,062	9,548	779,161
No. of resistance genes	3,628	839	109	159	70	21	15	4,841
No. of CAZymes	3,740	1,716	158	9,107	2,217	339	263	17,540
No. of N-cycling genes	354	210	46	2,601	556	141	42	3,950

a Excludes partial gene calls.

b Percentage of plasmids that contain at least one MOB gene.

10.1128/mbio.03191-22.1 FIG S1 Taxonomic representation of host bacteria in the PLSDB data set from the phylum to the genus level. More frequent bacterial taxa are represented by thicker lines. Line colors distinguish the phylum levels. Download FIG S1, PDF file, 2.4 MB. Copyright © 2023 Finks and Martiny. 2023 Finks and Martiny. https://creativecommons.org/licenses/by/4.0/ This content is distributed under the terms of the Creative Commons Attribution 4.0 International license.

10.1128/mbio.03191-22.2 FIG S2 Taxonomic representation of plasmid host phyla by environment in the PLSDB data set. Note that plasmid host phyla with a <1% relative abundance are not shown, e.g., plasmids from *Elusimicrobiota* (*n* = 3). Download FIG S2, PDF file, 0.2 MB. Copyright © 2023 Finks and Martiny. 2023 Finks and Martiny. https://creativecommons.org/licenses/by/4.0/ This content is distributed under the terms of the Creative Commons Attribution 4.0 International license.

### Plasmid properties vary by environment.

**FIG 1:**
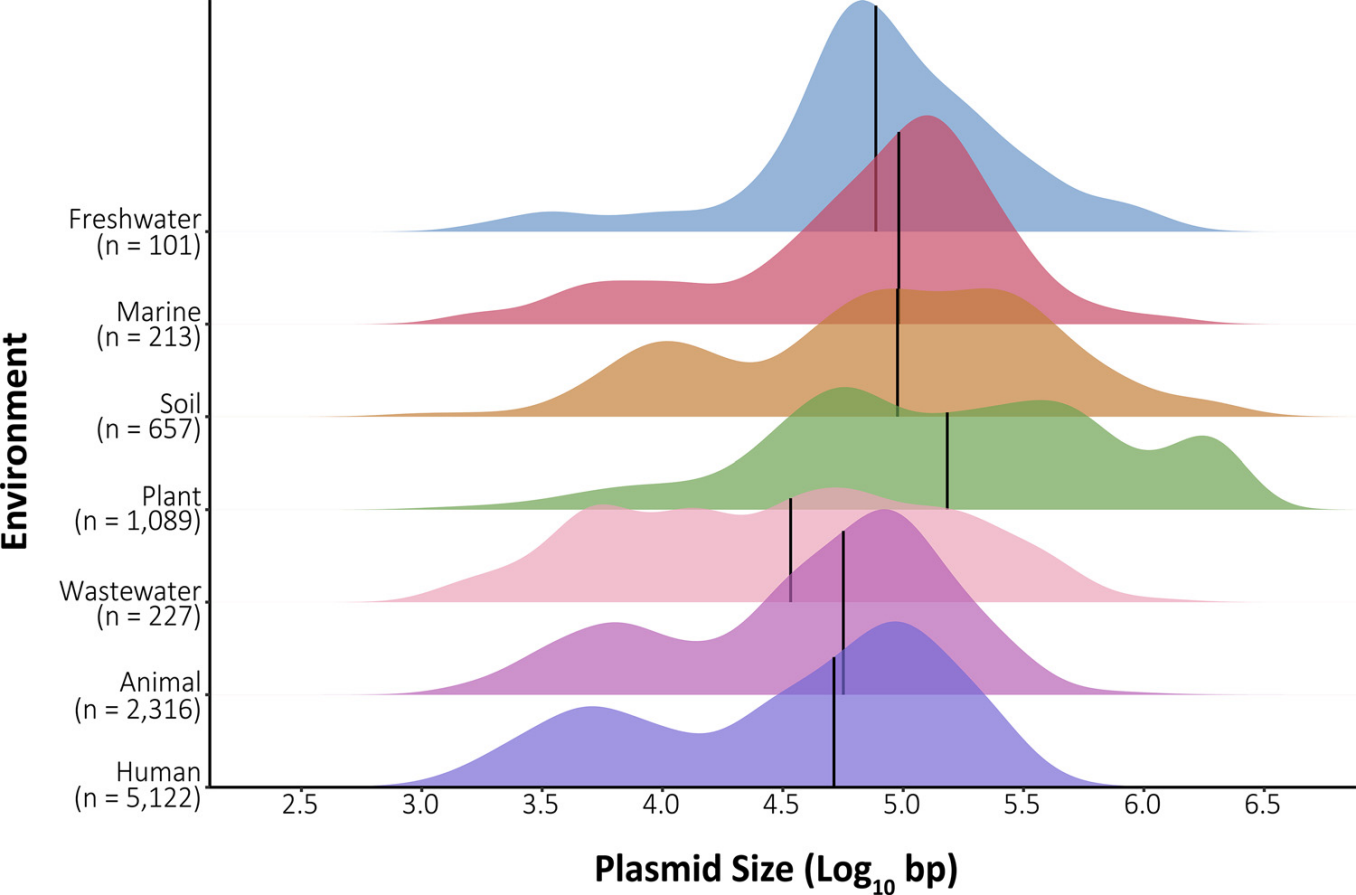
Plasmid size distributions vary significantly by environment. Shown are density plots of plasmid nucleotide lengths by environment. The mean plasmid sizes are indicated by vertical black lines. Untransformed mean plasmid sizes are 76.3 kb for human, 79.3 kb for animal, 88.4 kb for wastewater, 426.9 kb for plant, 215.5 kb for soil, 130.1 kb for marine, and 146.6 kb for freshwater environments.

Plasmid properties, including size, mobility genes, and plasmid replicon (incompatibility/replicase) types, differed by environment. Overall, plasmid sizes varied by environment [*H*(6) = 762.23; *P < *0.001 (by a Kruskal-Wallis test)] (Fig. 1), with plasmids isolated from plant and marine environments being larger than those from human and wastewater environments. Plasmid sizes also varied by phylum [*H*(12) = 521.9; *P < *0.001] (Fig. S3A), where the mean sizes of *Proteobacteria* plasmids were significantly larger (146 kb) than those of *Bacteroidetes*, *Firmicutes*, and *Chlamydiae* plasmids (73 kb, 68 kb, and 7.5 kb, respectively) (*P < *0.001 [by Wilcoxon tests]). However, plasmid size differences between environments were not entirely attributable to phylum composition as plasmid sizes differed across environments within phyla. For instance, the plasmid size within *Proteobacteria* (the most abundant host phylum in the data set) also differed by environment [*H*(6) = 717.99; *P < *0.001] (Fig. S3B).

10.1128/mbio.03191-22.3 FIG S3 Plasmid size distributions vary significantly by host taxonomy and environment. Shown are density plots of log_10_-transformed plasmid nucleotide lengths across 10 phyla (A) and across environments within *Proteobacteria* (B). The mean plasmid sizes are represented by vertical black lines at the center of the respective density distributions. Plasmid size distributions for phyla having <10 representatives are not shown. Untransformed mean plasmid sizes are as follows: 140.1 kb for *Actinobacteria*, 73.1 kb for *Bacteroidetes*, 7.5 kb for *Chlamydiae*, 128 kb for *Cyanobacteria*, 225 kb for *Deinococcus-Thermus*, 68 kb for *Firmicutes*, 21.2 kb for *Fusobacteria*, 69.4 kb for *Planctomycetes*, 146 kb for *Proteobacteria*, 33.9 kb for *Spirochaetes*, and 32.7 kb for *Tenericutes*. Likewise, the mean sizes of *Proteobacteria* plasmids by environment are as follows: 85.3 kb for human, 85.4 kb for animal, 81.5 kb for wastewater, 492.4 kb for plant, 284.1 kb for soil, 139.6 kb for marine, and 190.4 kb for freshwater. Download FIG S3, PDF file, 0.1 MB. Copyright © 2023 Finks and Martiny. 2023 Finks and Martiny. https://creativecommons.org/licenses/by/4.0/ This content is distributed under the terms of the Creative Commons Attribution 4.0 International license.

**FIG 2:**
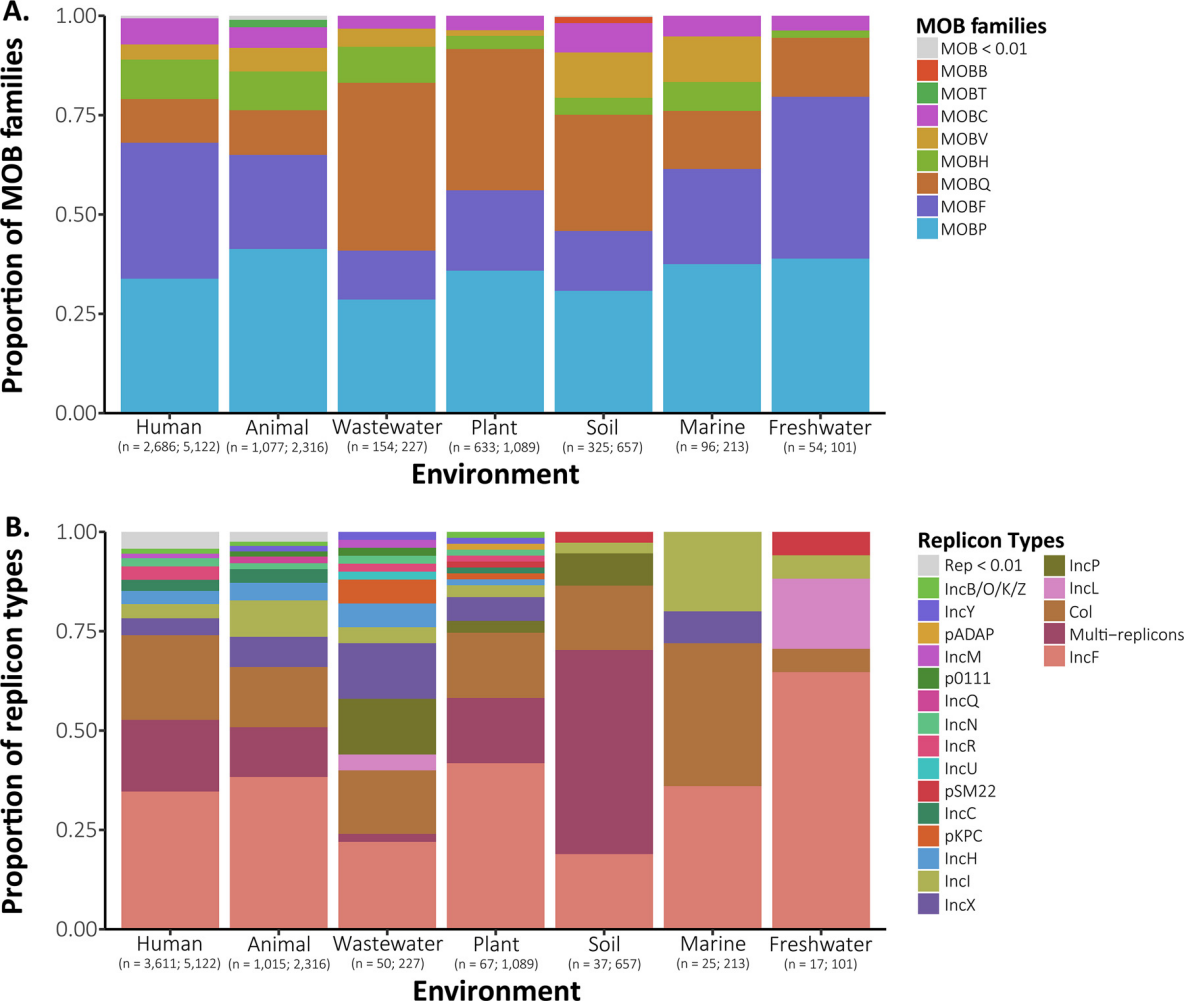
Proportions of plasmid (A) and replicon (B) types across environments. The values under each environment reflect the numbers of MOB/replicon types identified, followed by the total number of plasmids within an environment. MOB and replicon types represented by a <1% relative abundance within an environment are shaded in gray.

The most abundant genes encoding plasmid mobility (specifically, the MOB family of relaxases) were MOBP, MOBB, and MOBF genes (Fig. 2A). Moreover, the percentage of mobilizable plasmids (those having at least one MOB identified) ranged from 41 to 60% across environments, with the lowest percentage being found in marine environments and the highest being found in wastewater environments (Table 1). The MOB composition also varied by host phylum (*P* = 0.001 [by permutational multivariate analysis of variance {PERMANOVA}]) but not by environment (Fig. S4). Overall, 44% of the variation in MOB composition was explained by bacterial host phylum (Table S1), although we note that these differences could be due to differences in average composition and/or within-phylum variance (*P* = 0.004 [by permutational test for homogeneity of multivariate dispersions {PERMDISP}]) (49).

10.1128/mbio.03191-22.4 FIG S4 Proportion of relaxases by plasmid host taxonomy. The values under each plasmid host phylum (*n*) reflect the numbers of MOB relaxases identified (by family), followed by the total number of plasmids within a phylum. MOB families representing a <1% relative abundance within an environment are shaded in gray. Note that MOBP encompasses MOBP1-, MOBP2-, and MOBP3-type relaxases. Download FIG S4, PDF file, 0.2 MB. Copyright © 2023 Finks and Martiny. 2023 Finks and Martiny. https://creativecommons.org/licenses/by/4.0/ This content is distributed under the terms of the Creative Commons Attribution 4.0 International license.

10.1128/mbio.03191-22.7 TABLE S1 Results of PERMANOVA testing the effects of environment and host phylum (taxonomy) by trait type. Download Table S1, PDF file, 0.1 MB. Copyright © 2023 Finks and Martiny. 2023 Finks and Martiny. https://creativecommons.org/licenses/by/4.0/ This content is distributed under the terms of the Creative Commons Attribution 4.0 International license.

We also compared the plasmid replicon types provided by the PLSDB; 50% of the plasmids (*n* = 4,822) were assigned to a known replicon type (Fig. 2B). The most abundant replicon types across all environments were plasmids belonging to IncF- and colicin-type plasmids. Replicon types also varied significantly by environment [*G*(162) = 448.28; *P < *0.001], with distinct prevalences across some environments (Fig. S5), although most of the replicon type diversity (96% of the replicon types identified) was encompassed by plasmids from human and animal environments (Fig. 2B).

10.1128/mbio.03191-22.5 FIG S5 Plasmid replicon types by environment. The normalized frequencies of replicon types by environment are shown. To standardize for uneven plasmid sequences across environments, replicon types were first converted into proportional abundances within an environment after the removal of replicon types identified <6 times across all environments. Replicon types across environments were then normalized using Z-scores. Replicon types with above-mean (white tiles) values are represented by tiles shaded in red, while those with lower-than-mean values are shaded in blue. Download FIG S5, PDF file, 0.2 MB. Copyright © 2023 Finks and Martiny. 2023 Finks and Martiny. https://creativecommons.org/licenses/by/4.0/ This content is distributed under the terms of the Creative Commons Attribution 4.0 International license.

### Plasmid accessory traits vary by environment.

**FIG 3:**
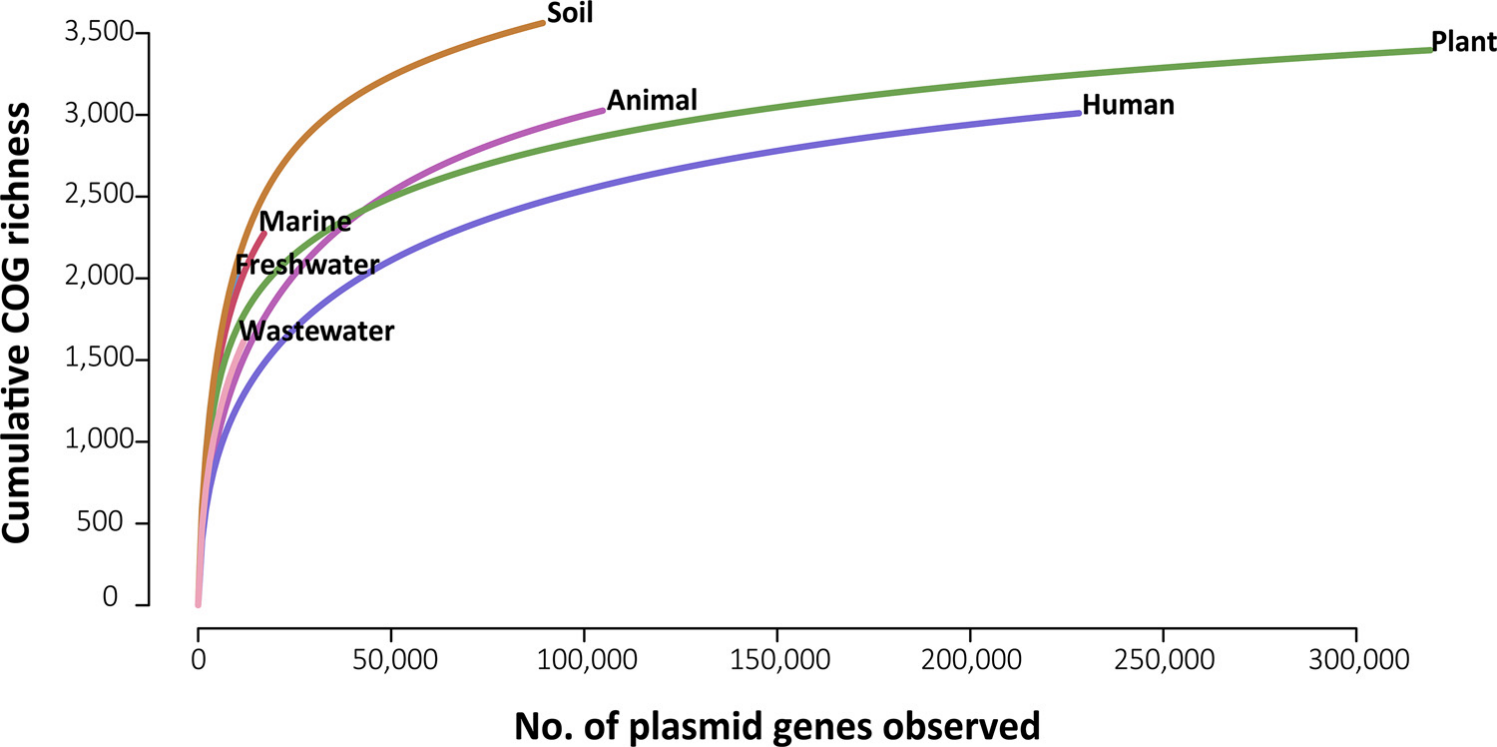
Cumulative COG richness of plasmids by environment. Unique COG function accessions were iteratively and randomly subsampled (*n* = 1,000 times) across each environment.

Overall, 56% of the plasmid gene calls were assigned to COG functions (Table 1). After standardizing for sampling differences among environments, plasmids from soil encompassed the highest functional diversity, and those from humans encompassed the lowest (Fig. 3). However, despite the large number of genes identified, the functional diversity remained undersampled for some environments; the identification of new COG functions increased steeply with each plasmid gene observed in aquatic, marine, and wastewater environments (Fig. 3).

**FIG 4:**
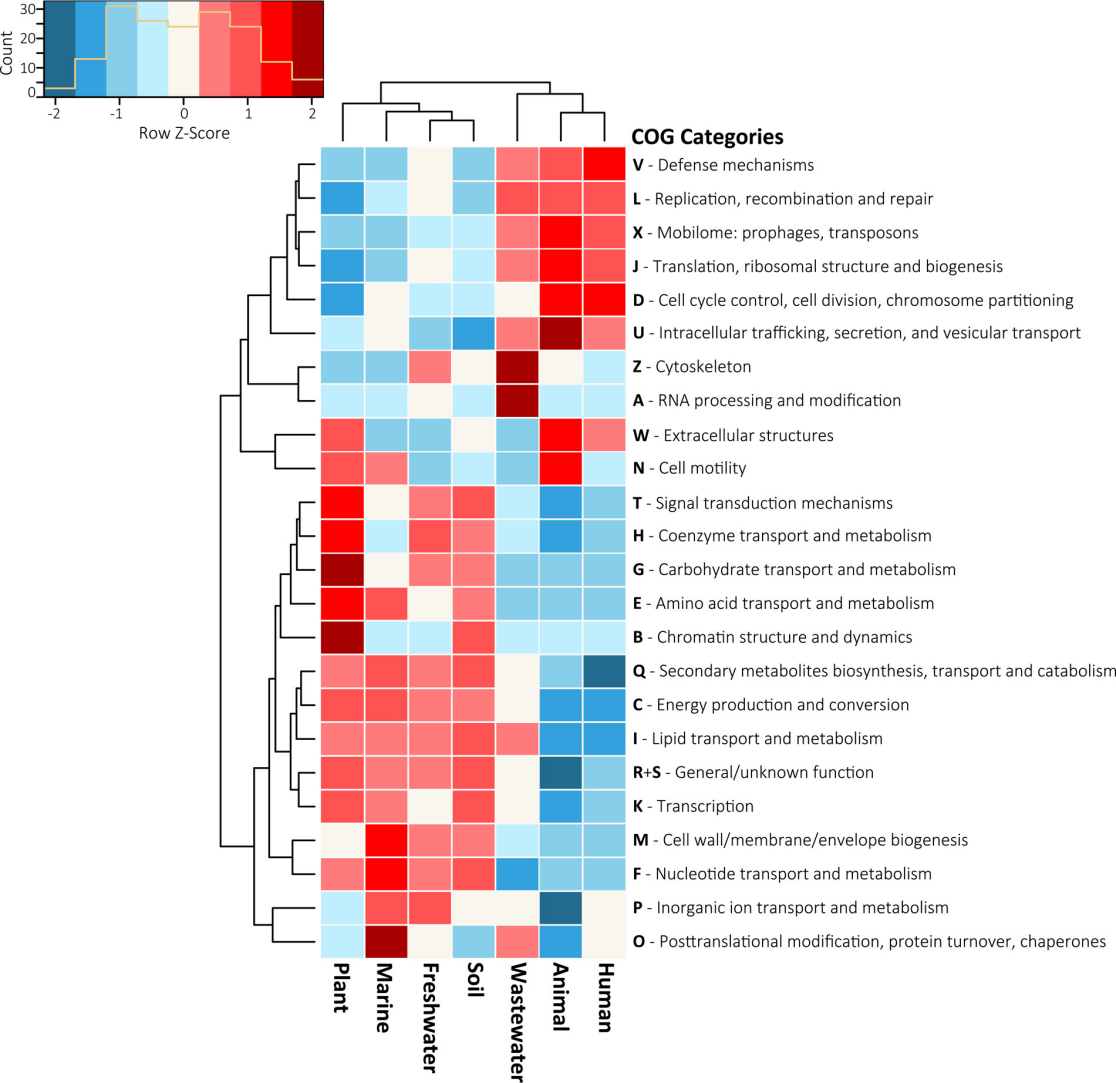
Plasmid COG functions by environment. Shown are the normalized frequencies of COG functions by environment at broader COG category designations. To standardize for uneven plasmid sequences across environments, COG function counts were first converted into proportional abundances within an environment after the removal of COG functions identified <6 times across all environments. COG abundances across environments were then normalized using Z-scores. COG categories with above-mean (white tiles) values are represented by tiles shaded in red, while those with lower-than-mean values are shaded in blue.

To investigate if accessory gene diversity varied by environment, we first considered broad COG categories (assigned A through X) of the plasmid genes. Indeed, the composition of COG categories differed significantly by environment [*G*(138) = 140,898; *P < *0.001 (by a *G* test)] (Fig. 4). Most COG categories were detected on plasmids across all environments but displayed distinct trends in their relative abundances. For example, COG categories of replication (L), defense mechanisms (V), recombination and repair (L), and the mobilome (X) were more prevalent in plasmids from wastewater, animal, and human environments. In contrast, COG categories for carbohydrate/amino acid/nucleotide transport and metabolism (G/E/F) and transcription (K) were more prevalent on plasmids from plant, marine, freshwater, and soil environments.

**FIG 5:**
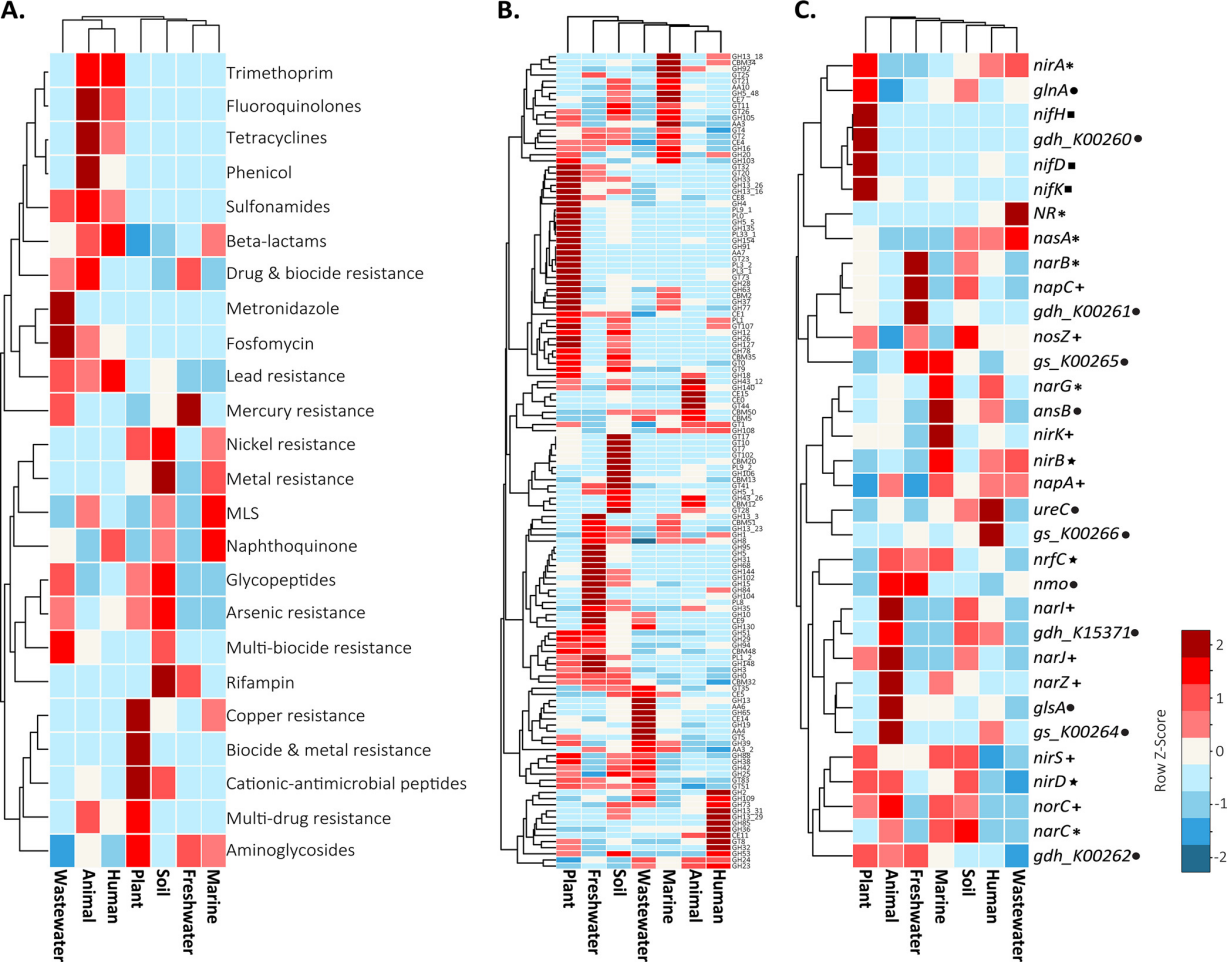
Plasmid resistance, carbon degradation, and nitrogen-cycling traits by environment. Shown are the normalized frequencies of resistance genes by classes (A), CAZyme family types (B), and N gene families (C) by environment. The resistance, CAZyme, and N gene families with above-mean (white tiles) values are represented by tiles shaded in red, while those with lower-than-mean values are shaded in blue. MLS, resistance genes within the classes that cover macrolide-lincosamide-streptogramin B. Symbols next to N gene family names represent the corresponding N-cycling pathways (asterisks, assimilatory nitrate reduction pathways; pluses denitrification; stars, dissimilatory nitrate reduction; squares, nitrogen fixation; circles, organic degradation and synthesis).

More than 4,800 genes encoding resistance were identified on the plasmids, but this number still accounted for <0.5% of all plasmid genes (Table 1). Of these genes, the majority conferred resistance to antibiotics (58%), followed by resistance to heavy metals and biocides (38%) and antimicrobials (4%). As with the broad COG categories, the composition of resistance genes also differed significantly by environment [*G*(138) = 1,058.9; *P < *0.001 (by a *G* test)] (Fig. 5A). Not surprisingly, resistance genes associated with commonly prescribed classes of antibiotics (trimethoprim, fluoroquinolones, tetracyclines, and phenicol) were relatively more prevalent in plasmids from animal and human environments. In contrast, plasmid resistance genes for heavy metals and biocides (metal, nickel, arsenic, and copper) were generally more prevalent in plant, soil, freshwater, and marine environments, with the exception that lead resistance genes were relatively more abundant in human, animal, and wastewater environments.

Genes encoding CAZymes represented 1.4% of the plasmid genes identified. Glycoside hydrolases (GHs) and glycosyltransferases (GTs) were the most common, accounting for 53% and 31% of the CAZymes identified (*n* = 9,367 and *n* = 5,497), respectively. As with the other accessory traits, CAZymes were present across environments but varied distinctly in their relative abundances [*G*(786) = 10,335; *P < *0.001] (Fig. 5B). For instance, GH5 and GH32 were highly prevalent in plasmids from plants, while GH2 and GH23 (19% of all CAZymes identified) were most prevalent in plasmids from human environments.

Nitrogen gene families represented <0.5% of the plasmid accessory traits identified but were nearly as abundant on plasmids as resistance genes (Table 1). The majority of these genes were assigned to two N-cycling pathways: organic degradation and synthesis (59%) and denitrification (22%). Like other accessory traits, N gene compositions varied by environment [*G*(192) = 1,049; *P < *0.001] (Fig. 5C). For instance, *napC* and *nirK*, encoding denitrification functions, were highly prevalent in freshwater and marine environments, respectively. In contrast, the nitrogenase gene *nifH* and the glutamate dehydrogenase gene *gdh_K00260* were identified only in plant environments. Of note, N gene families encoding nitrification and anaerobic ammonium oxidation were rarely identified or not detected on plasmids in this data set.

### Plasmid accessory traits vary by host phyla.

Given that the bacterial community composition varies tremendously across environments, the distribution of plasmid accessory traits could be driven largely by changes in host composition rather than by direct selection on the plasmid traits themselves. We hypothesized that plasmid traits would vary more strongly with environment than with bacterial host taxonomy, as many plasmids have the ability to be horizontally transferred and, thereby, break up a phylogenetic signal of the host. Indeed, the plasmid accessory trait composition varied significantly by host phylum for all trait types; however, the percent variance explained by host taxonomy ranged widely depending on the trait type (Table S1). In particular, host phylum explained 40% of the compositional variation in COG category traits, whereas it explained much less of the variation in resistance (13%), carbon utilization (1%), and nitrogen-cycling (10%) trait compositions. When controlling for host phylum, 9% of the variance in COG composition was explained by environment (*P* = 0.04 [by PERMANOVA]), and similar trends by environment were apparent for the COG categories among the three most abundant phyla (Fig. S6A). The composition of resistance genes, CAZyme genes, and N gene families within phyla did not vary significantly across environments, although this result is likely due to the undersampling of these specific traits within *Firmicutes* and *Actinobacteria* (Fig. S6B to D).

10.1128/mbio.03191-22.6 FIG S6 Plasmid accessory traits by environment and the three most abundant bacterial phyla. (A) Plasmid COG categories. The counts are standardized and normalized as described in the text. (B) Plasmid resistance traits by environment for *Proteobacteria* and *Firmicutes*. The resistance genes are grouped by resistance class. Note that resistance genes of *Firmicutes* from freshwater environments are not shown due to their low frequencies (<15 genes). (C) Plasmid carbohydrate utilization genes encoding CAZyme families. (D) Plasmid nitrogen-cycling genes. MLS, resistance genes within the classes that cover macrolide-lincosamide-streptogramin B. The symbols at the right of the N gene family names correspond to the different N-cycling pathways. For all heat maps, gray tiles represent undetected genes for the corresponding traits. Download FIG S6, PDF file, 2.5 MB. Copyright © 2023 Finks and Martiny. 2023 Finks and Martiny. https://creativecommons.org/licenses/by/4.0/ This content is distributed under the terms of the Creative Commons Attribution 4.0 International license.

### Mobility potential of plasmid accessory traits.

We hypothesized that some accessory traits, those that might confer a sudden and strong selective advantage, such as resistance traits, would be more frequently associated with mobilizable plasmids. Approximately one-half of all accessory traits were identified in plasmids having at least one MOB family relaxase (required for both conjugative and mobilizable plasmids). The highest percentages of potentially mobilizable genes (on a plasmid with a MOB gene) were identified in resistance genes (58%), compared to the lowest in nitrogen cycling genes (32%) (Table 2). Furthermore, plasmids from human, animal, and wastewater environments had higher percentages of mobilizable traits than the other environments (e.g., 58.9% of plasmid COGs from human versus 31.6% from soil environments) [*G*(6) = 26,449; *P < *0.001], except for N-cycling traits. In that case, plasmids from plant environments had the highest percentage (53.6%) of mobilizable N gene families.

**TABLE 2:** Summary of traits associated with mobilizable^*a*^ plasmids by environment

Trait by plasmid MOB count	Value for environment							
	Avg	Human	Animal	Wastewater	Plant	Soil	Marine	Freshwater
COGs								
No. mobilizable		134,125	48,610	6,650	133,044	28,227	6,073	4,821
No. nontransmissible		93,643	56,054	5,092	186,094	61,012	10,989	4,727
% mobilizable	45.9	58.9	46.4	56.6	41.7	31.6	35.6	50.5
Resistance								
No. mobilizable		2,380	563	58	68	24	15	11
No. nontransmissible		1,248	276	51	91	46	6	4
% mobilizable	58.2	65.6	67.1	53.2	42.8	34.3	71.4	73.3
CAZymes								
No. mobilizable		2,582	850	89	3,630	603	111	108
No. nontransmissible		1,158	866	69	5,477	1,614	228	155
% mobilizable	45.2	69.0	49.5	56.3	39.9	27.2	32.7	41.1
N gene families								
No. mobilizable		114	66	11	1,394	137	37	14
No. nontransmissible		240	144	35	1,207	419	104	28
% mobilizable	32.2	32.2	31.4	23.9	53.6	24.6	26.2	33.3

a Mobilizable indicates a plasmid having at least one MOB gene identified.

The distribution of potentially mobilizable traits also varied widely and significantly within specific traits (Tables S2A to C) (all *P < *0.001 [by *G* tests]). For instance, over 65% of the plasmid genes encoding resistance to the β-lactam, naphthoquinone, and sulfonamide drug classes were associated with mobilizable plasmids, compared to those encoding resistance to the rifampin and tetracycline classes, with <50% (Table S2B). Similarly, GH23 and PL3_1 CAZymes were more often associated with mobilizable plasmids (80% and 90%, respectively) than GH1 or PL9_2 (39% and 5% mobilizable, respectively) (Table S2C). Nitrogen-fixing genes (*nifD*, *nifH*, *nifK*, and *nifW*) were associated with the highest percentages of mobilizable plasmids (93%, 89%, 85%, and 100%, respectively), compared to genes encoding denitrification pathways, such as *napA* and *napC* (33% and 44%, respectively) (Table S2D).

10.1128/mbio.03191-22.8 TABLE S2 (A) Summary of COG categories associated with mobilizable plasmids (those having at least one detected relaxase). (B) Summary of resistance genes associated with mobilizable plasmids. (C) Summary of CAZymes by family associated with mobilizable plasmids. (D) Summary of N gene families associated with mobilizable plasmids. Download Table S2, PDF file, 0.2 MB. Copyright © 2023 Finks and Martiny. 2023 Finks and Martiny. https://creativecommons.org/licenses/by/4.0/ This content is distributed under the terms of the Creative Commons Attribution 4.0 International license.

### Trait variation is not entirely explained by taxonomic differences.

The above-described results demonstrate that the accessory gene content varied across environments even within the most abundant phyla in the data set. To further test the sensitivity of the results to host compositional differences among environments, we tested whether the accessory gene content varied by environment within the most abundant host species (Escherichia coli [19% of all plasmids]). The bulk of the E. coli plasmids were isolated from animal and human environments (36% and 61%, respectively), and no representatives were isolated from soil. However, the COG composition varied significantly between human and nonhuman animal environments [*G*(22) = 58.8; *P < *0.001 (by a *G* test)]. Likewise, the contents of antibiotic resistance genes, carbohydrate utilization and nitrogen-cycling traits, and plasmid mobility genes on E. coli plasmids significantly differed across humans and animals (*P < *0.001 for all tests).

We also removed the three most abundant genera (Acinetobacter, Escherichia, and Klebsiella) from *Proteobacteria* and the most abundant genus (Staphylococcus) from *Firmicutes* and tested that the results held within these phyla. Among the 3,330 remaining *Proteobacteria* plasmids, the overall COG composition, resistance genes, carbon and nitrogen utilization traits, and plasmid mobility genes varied significantly across the seven environments. Similarly, all traits differed by environment among the 1,028 remaining *Firmicutes* plasmids (*P < *0.001 for all tests).

## DISCUSSION

We identified a high level of diversity of accessory genes on plasmids from a large public database. Although most plasmid research focuses on resistance traits, such genes made up only a small fraction (0.4%) of the total genetic information carried by the plasmids in this database. Genes associated with nitrogen cycling were nearly as common, and those associated with carbohydrate degradation were an order of magnitude more abundant (1.4%). In contrast to reports of environmental plasmids that carry few accessory genes (50), the abundance and diversity of accessory genes on plasmids from across all environment types are remarkable.

Plasmids may confer traits that allow a bacterial host to adapt to its local environment. In support of our first hypothesis, we found that the compositions of these genes varied significantly by environment. These cross-environment differences do not appear to be entirely driven solely by differences in microbial compositions across environments or by a few very abundant groups, for at least three reasons. First, the patterns held for a variety of taxonomic subsets of the data, comparing within phyla, within phyla after removing the most abundant genera, and within the most abundant species, E. coli. Second, plasmid-encoded accessory traits seem to partially reflect their environment, similar to the traits encoded on the bacterial chromosome. For instance, the CAZyme compositions on plasmids varied across environments, just as the patterns of CAZymes from whole genomes also vary (51). Third, our results support those of a variety of more-focused studies that have previously noted differences in plasmid traits among similar types of environments (48, 52–55). Thus, despite the limitations of the data set, we conclude that plasmid accessory genes vary across the seven broadly defined environments studied here.

Notably, plasmid accessory traits seem to form two larger groupings by environment: human/animal/wastewater and plant/soil/marine/freshwater environments. This clustering could be due to the greater bacterial dispersal of plasmids across some environments than across others. For instance, microbial communities of wastewater environments reflect the microbiomes of human populations through sewer systems and surface runoff (56). Alternatively, the clustering in accessory gene composition could be due to more similar selection pressures within the two environmental groupings.

Contrary to our second hypothesis, plasmid traits appear to vary more strongly by host taxonomy at the phylum level than by environment. This pattern held for both plasmid mobility (MOB genes) and accessory traits. Only the composition of COG functions was significantly affected by the environment after controlling for host phylum. Thus, despite high levels of HGT of plasmids within phyla (45, 57), a host’s evolutionary history seems to limit the types of traits carried by a plasmid. This result is in alignment with the results of recent work indicating that plasmids and their mobility traits are constrained by the evolutionary history of their hosts, even as some hosts are permissive to gene exchange beyond species/genus borders (45, 58, 59). It could also be that plasmid accessory genes are influenced by interactions among the genes themselves. Indeed, a recent analysis of metabolic genes on conjugative plasmids of E. coli demonstrated statistically significant associations and disassociations with known antibiotic resistance genes at the strain level, suggesting that each gene type may impact the spread of the others across hosts (60). We caution, however, that the analysis here gives an approximation; our ability to test the role of host phylogeny is limited by the uneven data available across the phylogeny and environments. In the future, it would be useful to investigate the changing influence of host phylogeny versus environment on plasmid traits as one moves from broader to finer taxonomic scales, where HGT may be more prevalent (61, 62).

Given that genes associated with plasmid mobility varied by environment, we next asked if plasmid accessory traits varied in their associations with these plasmid properties. In support of our last hypothesis, plasmid accessory traits varied in their associations with the presence of MOB genes but not by replicon type. As expected, resistance traits were found more often on potentially mobilizable plasmids than any other type of trait (63). The connections between plasmid traits and mobility also varied by environment; resistance traits appear more mobilizable in the human/animal/wastewater environments than any other trait in any other environment (with the exception of N genes in plant plasmids). This pattern may be due to culturing bias; however, stable temperatures and resources, along with high concentrations of bacterial cells, may make human environments especially favorable for HGT (64). A caveat of our analysis is that the presence of MOB genes does not indicate the potential for plasmid transmissibility or actual HGT frequency (65). For instance, there may be unrecognized mechanisms of plasmid transfer, particularly in less-well-studied environments. Indeed, nontransmissible plasmids appeared to be widely disseminated among members of a *Vibrionaceae* population despite the lack of a clear mechanism of transmission (66).

### Conclusions.

A key aspect of plasmid diversity and evolution is the relationship between plasmids, their bacterial hosts, and the environment. Our results suggest that plasmid-bound traits offer a substantial source of genetic diversity for bacterial adaptation to their particular environment, advancing our understanding of the fate of plasmids (67). However, more work is needed to directly link these mobile genetic elements to host adaptation in natural communities. Notably, not all plasmids are expected to carry accessory genes (68–70); further investigation of these cryptic plasmids may reveal additional insights into forces maintaining them in natural communities. While we focused on plasmid sequences from cultured microbes, this approach is limited by the available data but links plasmid traits directly with the host, an advantage that culture-independent plasmidome studies do not have. Thus, implementing sequencing approaches like Hi-C (71) in future plasmidome studies could also help address this disconnect between plasmids and their host, which has shown promise in some studies connecting the resistome and plasmidome to wastewater microbiomes (27). Additionally, by combining current methods in novel ways, such as cell enumeration and sorting with amplicon sequencing of microbial communities, plasmid fitness effects can be studied within microbial communities (72), thereby helping us to understand the adaptive role of plasmids. Furthermore, efforts to culture and sequence plasmids from underrepresented clades and environments, such as *Cyanobacteria* and *Actinobacteria* and aquatic environments, would be enormously valuable as 75% of the plasmid sequences analyzed here were from *Proteobacteria*. Finally, an outstanding question is the role of plasmids in microbial functioning at the community scale. Recent experiments demonstrate that manipulation of mobile genetic elements in communities may influence biogeochemical processes such as nitrogen cycling (73), pointing to exciting directions for investigating how plasmid evolution influences the ecology of microbial communities.

## MATERIALS AND METHODS

### Retrieval of plasmids from different environments.

Plasmid sequences and metadata were retrieved from the curated plasmid database PLSDB v.2020_06_29 containing 23,227 plasmids on 14 October 2020 (42). Plasmid sequences from seven environment types (human, animal/nonhuman, wastewater, plant, soil, marine, and freshwater) were obtained by using the following PLSDB-provided metadata: IsolationSource_BIOSAMPLE, Host_BIOSAMPLE, and SampleType_BIOSAMPLE. Plasmids from human environments were identified by the following search terms: human, OR Homo sapiens, OR child, OR patient. Plasmids from animal and plant environments were identified by common and/or scientific names (e.g., mouse, OR mice, OR Mus musculus). Plasmids from wastewater were identified by the search terms wastewater, OR sewage, OR sludge, while plasmids from soil, marine, and freshwater environments were identified by the terms soil(s), OR mud, OR permafrost; marine, OR ocean, OR sea, OR seawater, OR beach; and freshwater, OR lake, OR river, OR creek, OR stream, respectively. Plasmids identified using the search terms rhizosphere, OR root, OR root nodule were assigned as belonging to the plant environment, while sequences identified as “rhizosphere soil” were assigned to the soil environment. Duplicate plasmid sequences were removed using the unique plasmid record identifier UID_NUCCORE. If BioSample categories resulted in the same accessions being binned into different environments, the plasmid environment assignment was determined first by sample type, then by host (e.g., human or animal), and finally by isolation source. Plasmid sequences were retrieved from the PLSDB files using the UID_NUCCORE identifier and the blastcmd feature of BLAST+ v2.10.0 (74).

### Plasmid properties and accessory trait identification.

To identify plasmid sizes (nucleotide lengths), the Length_NUCCORE metadata of the PLSDB were used. Depending on the sequence format of the databases used for trait identification, different search tools were employed. Plasmid sequences were searched for MOB family relaxases, which are essential for conjugative DNA processing (6), using MobScan (https://castillo.dicom.unican.es/mobscan_about/) (75), after assigning gene calls using Prodigal v2.6.3 (76). MOB hits with per-domain thresholds (i-E values) of ≤1e−5 and >60% query coverages were included in the analysis. Since the PLSDB also runs plasmid sequences through the PlasmidFinder (77) and Plasmid Multilocus Sequence Typing (pMLST) schemes and uses profiles from PubMLST (78), we utilize the existing replicon typing (i.e., replicase/incompatibility group) information provided by the PLSDB for plasmid sequences included in this data set (42).

To identify pathway and functional systems encompassing a diversity of traits, the Clusters of Orthologous Genes database, release 2020 (79), was searched using plasmid gene calls (excluding partial gene calls) and DIAMOND v0.9.14 in sensitive mode (80). All COG hits with E values of ≤0 were included in the analysis, and in cases where multiple domains for a given gene call resulted in more than one COG function and category assignment, only annotations for the first domain hit were included in the downstream analyses. For identifying traits involved in biogeochemical processes (carbon and nitrogen-cycling pathways) and heavy metal and antibiotic resistance determinants, similarity searches of plasmid gene calls against the following databases were used: the standalone version of dbCAN2, release 2019-07-31 (81); the NCycDB, with curated nitrogen (N) gene family sequences (encompassing seven N-cycling pathways) at 100% sequence identity, release 2019 (82); and MEGARes version 2.0.0, a database for the classification of heavy metal, biocide, antimicrobial, and antibiotic resistance determinants (83). For carbohydrate utilization trait analyses, CAZyme hits in the dbCAN2 database were included if two or more of the three search tools (HMMER, DIAMOND, and Hotpep) matched in their identifications of the same CAZyme family. For instances where a single gene call returned multiple matches to CAZyme families, only the annotations from the first domain hit were included in downstream analyses. For N gene family hits, BLASTp searches of plasmid gene calls having E values of ≤10^−5^ and >50% query coverages were included in the analyses. Since N gene families encode a single N-cycling pathway in the NCycDB, these terms are used interchangeably throughout this paper. For resistance determinant identification, BLASTn searches of plasmid gene calls having E values of ≤10^−5^ and >85% query coverage, per subject and high-scoring pairs, were included in the analyses.

To determine the extent to which accessory traits might be mobilized via plasmids, we assigned potential plasmid mobility based on previous assessments (13). However, here, we do not distinguish self-transmissible/conjugative plasmids from mobilizable plasmids. Since known plasmid DNA relaxases (MOB) with or without genes encoding type IV coupling proteins are presumed mobilizable, while conjugative plasmids require the major components of a type IV secretion system (T4SS) in addition to MOB genes to be self-transmissible, we broadly define the potential of plasmids to move to other bacteria based on the presence or absence of known MOB genes and focus on the associated accessory trait distributions by environment. We refer the reader to previous studies for an in-depth review of the quantification and diversity of T4SSs and plasmid mobility (13, 84).

### Plasmid accessory trait and clustering analyses.

To standardize for uneven plasmid sequences across environments, trait counts were first converted into proportional abundances within an environment after the removal of rare traits (trait counts of <6 across all environments). Trait abundances across environments were then normalized using Z-scores in R v3.6.3 (85). This procedure served to weigh each trait similarly rather than proportionally to its abundance. To compare the similarities of traits across environments, we then calculated the Euclidean distance of the standardized and normalized trait count data using the vegdist function of the vegan package in R (86). To determine trait clustering by environment and trait category, agglomerative hierarchical clustering of the distance matrices was performed using the hclust function (clustering method, “average” for the unweighted pair group method using average linkages [UPGMA]) of the stats package in R. To visualize trait clustering results, heat maps using trait distance matrices were passed to the heatmap.2 function of the gplots package (https://github.com/talgalili/gplots) in R. To determine the extent to which total plasmid trait diversity is characterized across plasmid environments, cumulative COG richness was assessed for unique COG function accessions grouped into broader category designations, which were subsampled across each environment using the rarecurve function (step size = 1,000) of the vegan package in R.

### Statistical analysis.

To assess the differences in how plasmid size may vary by environment and host taxonomy (here, “host” refers to the bacteria from which plasmids were isolated and is distinct from host-associated environments, e.g., humans and animals), we used Kruskal-Wallis tests on the log_10_-transformed nucleotide lengths (base pairs) of plasmid sequences grouped by environment or host phylum using the stats package of R. *Post hoc* Wilcoxon tests (with Bonferroni-adjusted *P* values for multiple comparisons) of plasmid sizes by environment, phylum, and environment given a particular phylum were performed using the pairwise.wilcox.test function of the stats package in R. To disentangle the influence of environment and/or host taxonomy on plasmid trait composition, permutational multivariate analysis of variance (PERMANOVA) (*n *= 999 permutations under a reduced model) on Euclidean distance matrices of standardized and normalized trait counts, as mentioned above, was performed in PRIMER-e v6 (87–89). We used plasmid accessory traits in a fixed-effects model that included environment and host phylum, with no interaction terms, partial sums of squares, and fixed-effects sum to zero to test for the effect (significance and variance explained) of host taxonomy versus environment on the composition of each trait type. Since plasmids from some host taxa were represented across few environments (e.g., plasmids of *Chlamydiae* were present in human and animal environments only), we tested the influence of host phylum on the accessory trait content for plasmid host taxa with at least 50 representatives in the data set. The percentages of estimated variance explained for significant factors were determined by dividing the terms by the sum of the estimates of components of variation and multiplying by 100. To determine that the assumptions of PERMANOVAs were met, PERMDISP, a distance-based test for the homogeneity of multivariate dispersion, was performed in PRIMER-e (*n *= 999 permutations) to measure deviations from group centroids (87, 88). To determine whether the proportions of traits were different among environments, log-likelihood-ratio tests (*G* tests) of independence using the GTest function (with no correction) of the DescTools v0.99.44 package (https://andrisignorell.github.io/DescTools/) in R were performed. *G* tests were performed on contingency tables of nonstandardized trait counts, with rare traits (trait counts of <6 across all environments) removed. Stacked bar plots of standardized MOB gene counts by environment and linear plots of plasmid coding densities by environment were constructed using ggplot2 (https://ggplot2.tidyverse.org).

### Sensitivity analyses.

We also tested the sensitivity of the result that accessory gene contents differed by environment to sampling biases of the plasmid database by repeating our main analyses on subsets of the data. To do this, we first calculated the taxonomic distribution of bacterial hosts in the data set using the R package ggsankey (https://github.com/davidsjoberg/ggsankey). Next, we investigated patterns within the most abundant phyla, as explained above. We then examined trends within E. coli, the most abundant species represented. Finally, we removed the three most abundant genera from *Proteobacteria* (Acinetobacter, Escherichia, and Klebsiella) and the most abundant genus from *Firmicutes* (Staphylococcus). We then retested that the results held within these two dominant phyla.

### Data availability.

All scripts are accessible on GitHub (https://github.com/SaraiFinks). The plasmid sequences and metadata are available through the PLSDB (https://ccb-microbe.cs.uni-saarland.de/plsdb/).
