# Supplementary figures and images for: Plasmid-Encoded Traits Vary across Environments
Source: mBio. 2023 Jan 11;14(1):e03191-22. doi: 10.1128/mbio.03191-22 (PMC9973032; doi:10.1128/mbio.03191-22)

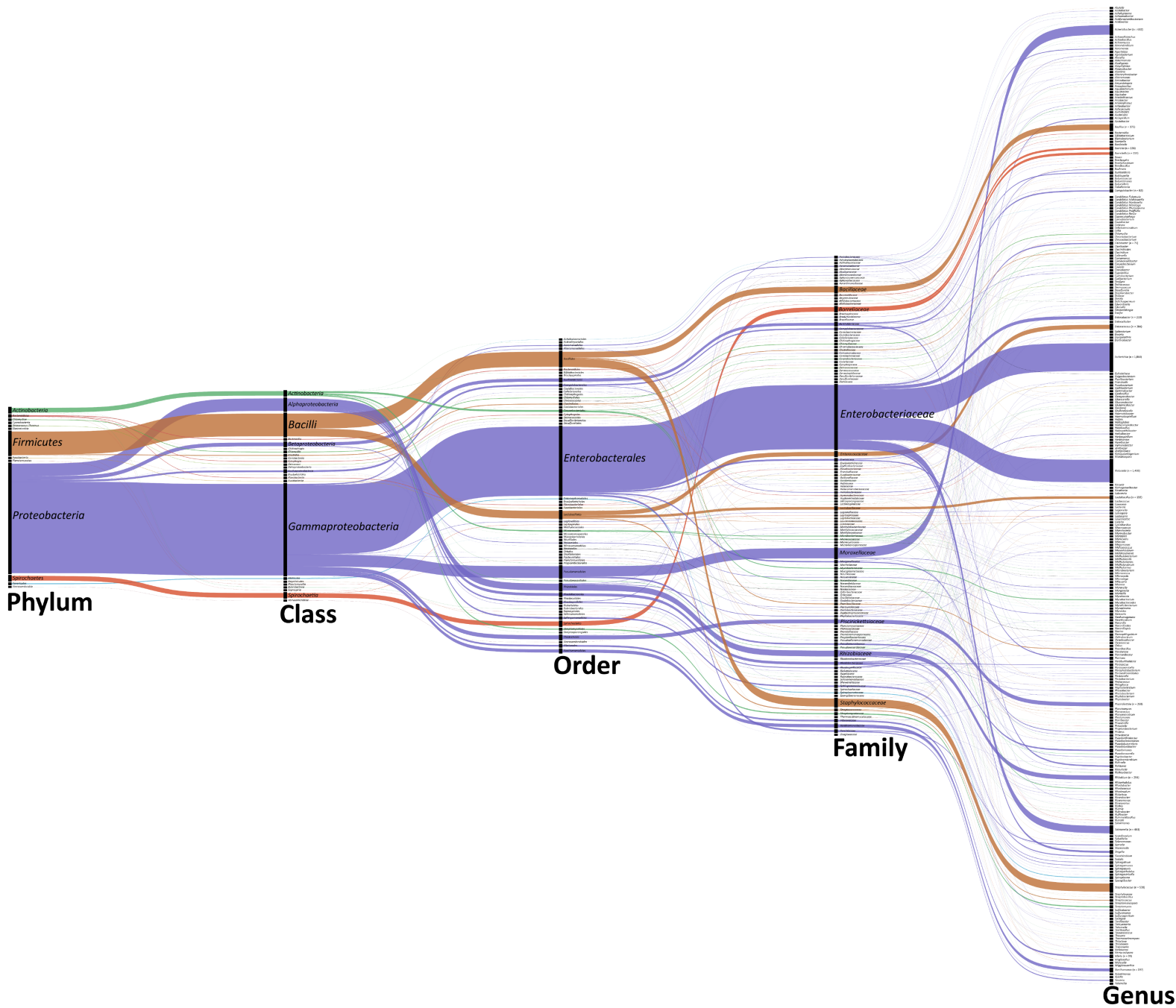

Supplement: FIG S1 [file mbio.03191-22-s0001.pdf]

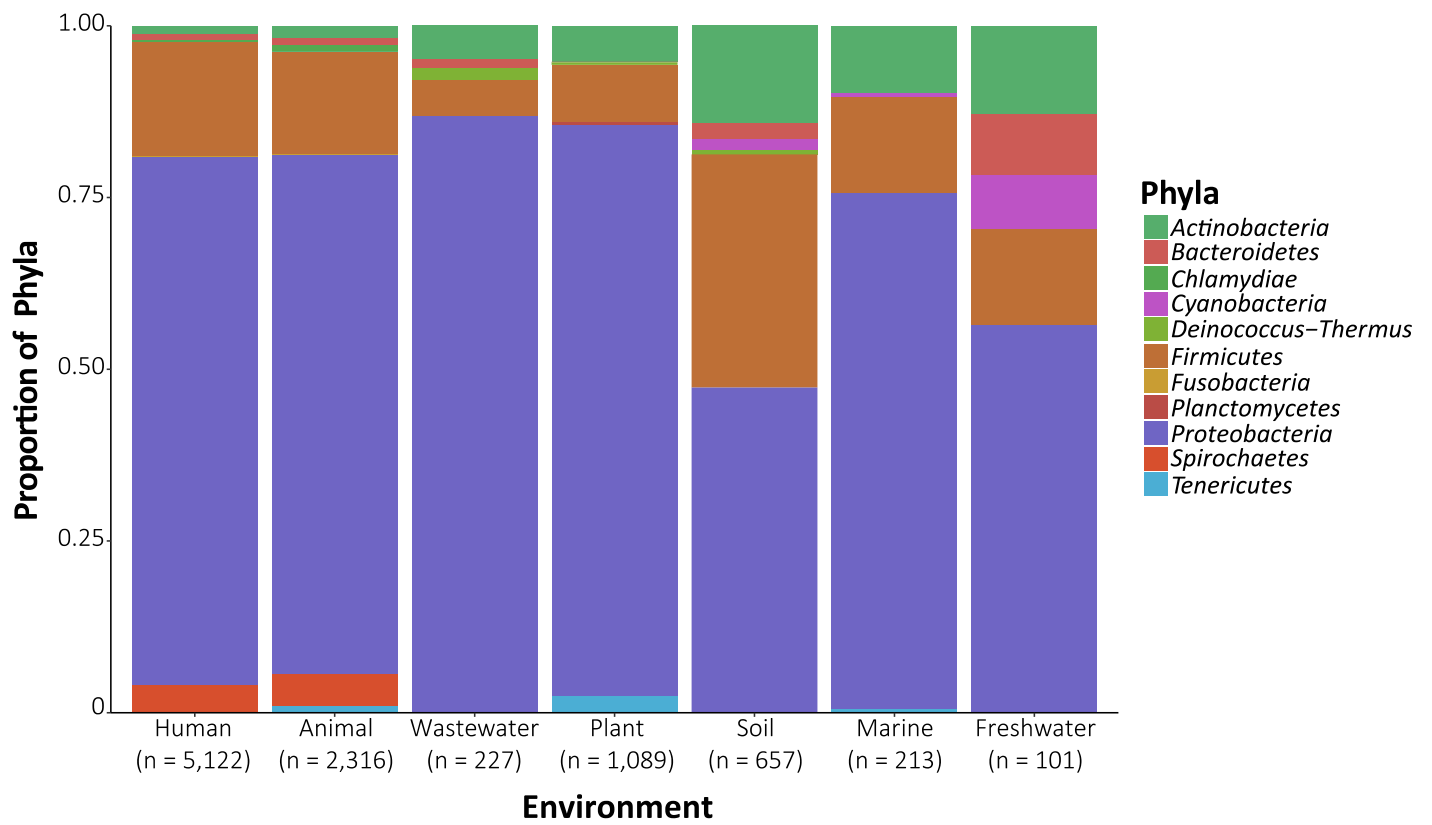

Supplement: FIG S2 [file mbio.03191-22-s0002.pdf]

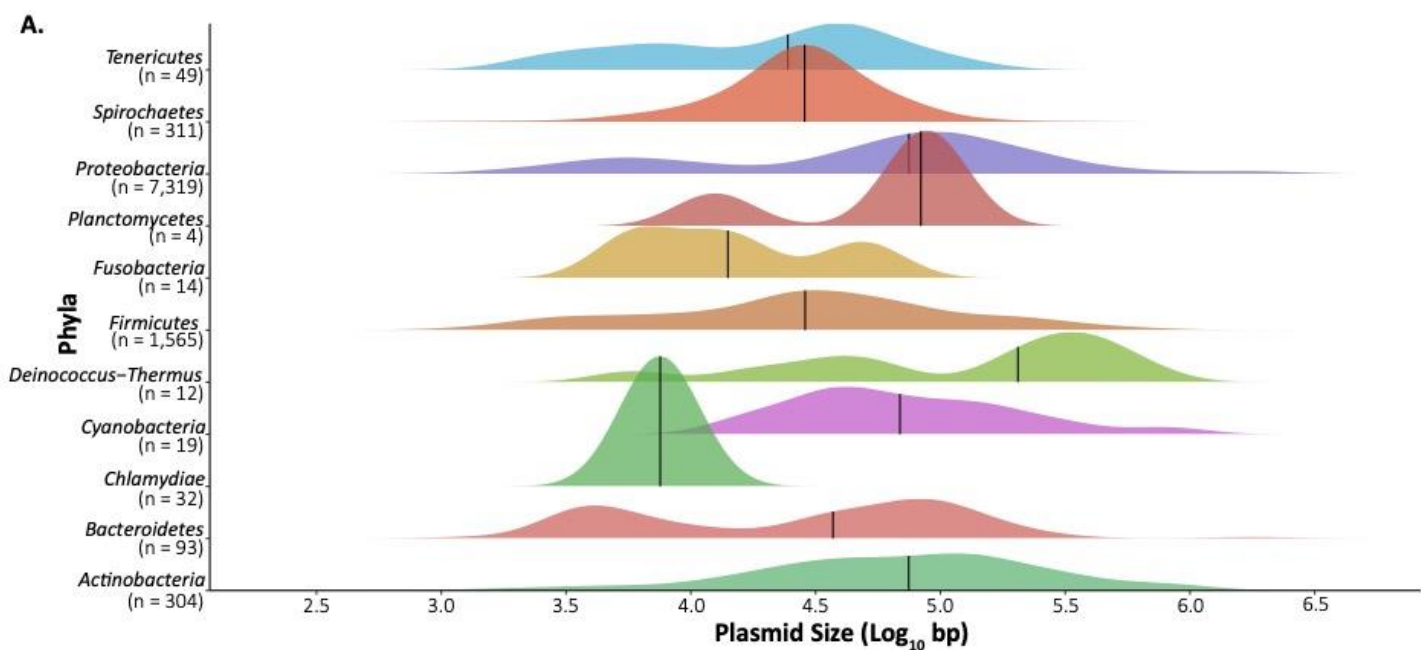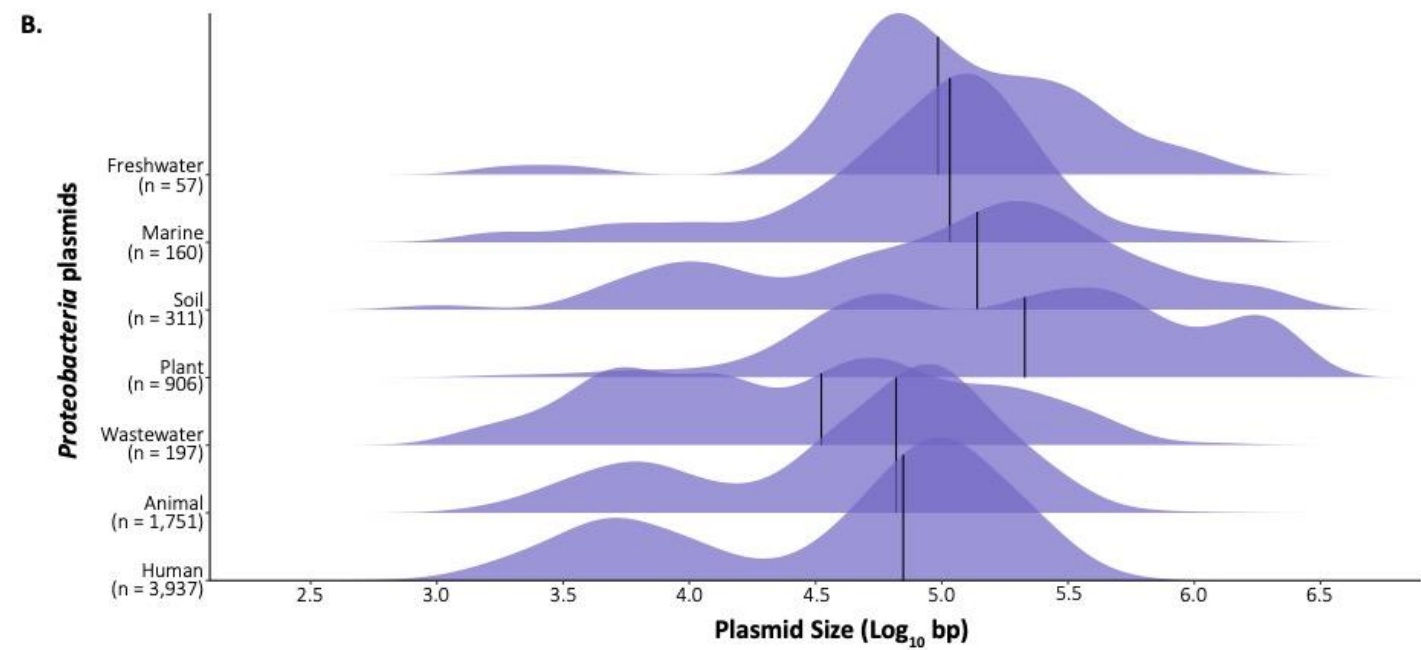

Supplement: FIG S3 [file mbio.03191-22-s0003.pdf]

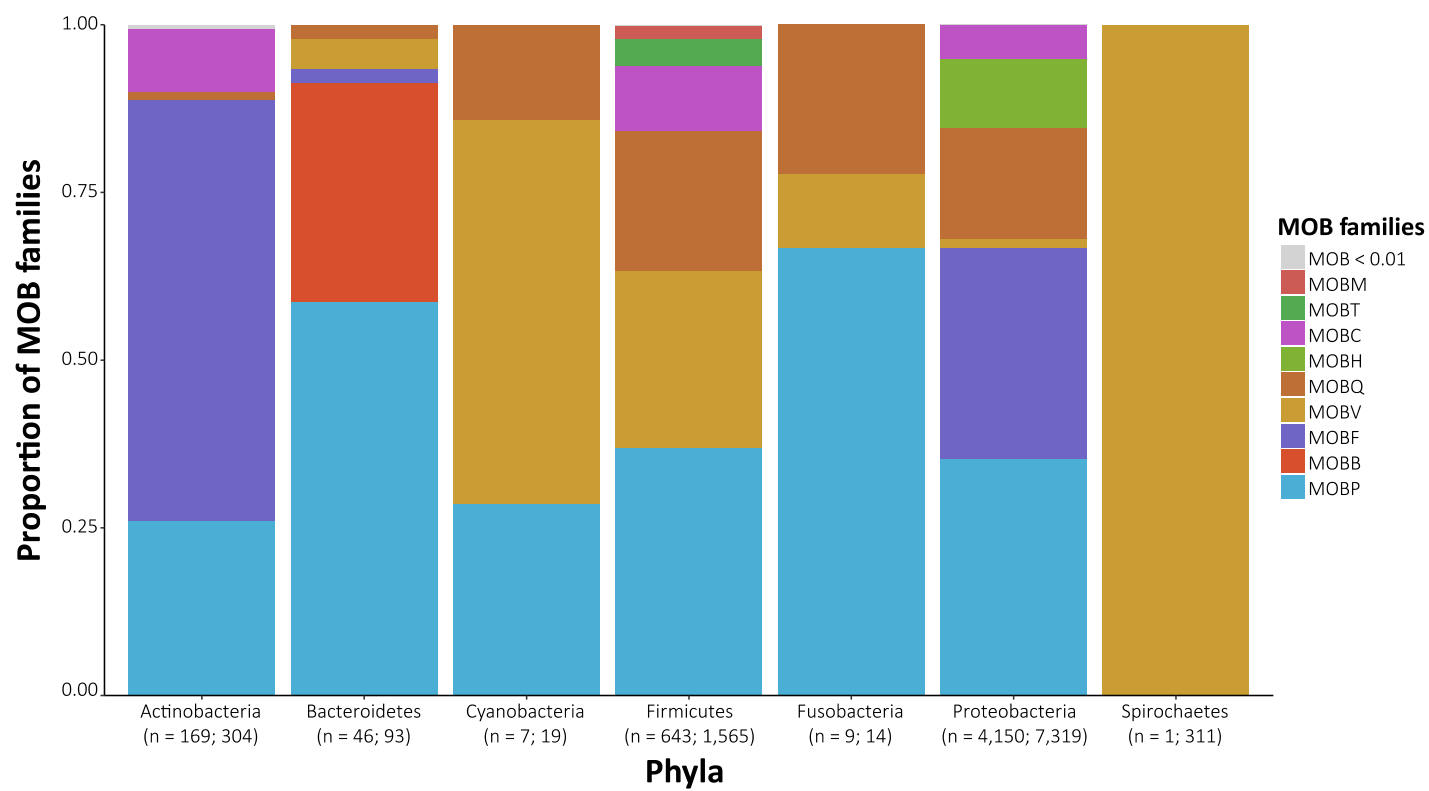

Supplement: FIG S4 [file mbio.03191-22-s0004.pdf]

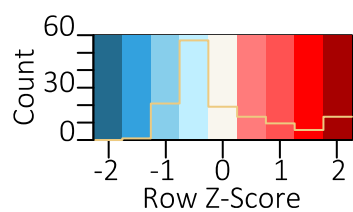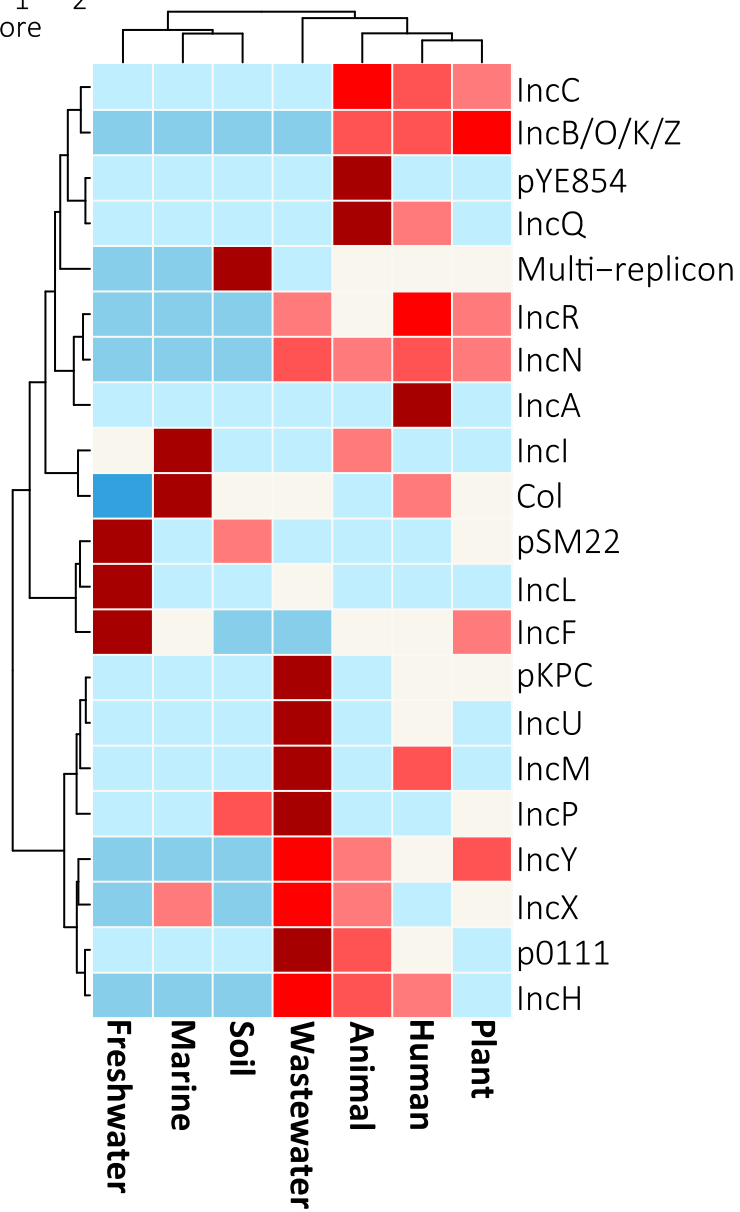

Supplement: FIG S5 [file mbio.03191-22-s0006.pdf]
