# Supplementary material for: Plasmid-Encoded Traits Vary across Environments
Source: mBio. 2023 Jan 11;14(1):e03191-22. doi: 10.1128/mbio.03191-22 (PMC9973032; doi:10.1128/mbio.03191-22)
Supplement: TABLE S1 [file mbio.03191-22-s0005.pdf]

Table S1. PERMANOVA testing the effects of environment and host phylum (taxonomy) by trait type*.

Trait/factors	DF	SS	MS	Pseudo-F	*P*-value	ECV (%)
**COG**						
Environment	6	123.38	20.56	1.58	**0.044**	9.3
Host Phyla	3	203.98	67.99	5.23	**0.001**	40.1
Residuals	13	169.04	13.00			50.6
**Resistance**						
Environment	6	124.93	20.82	0.92	0.710	-
Host Phyla	3	113.40	37.80	1.67	**0.008**	13.5
Residuals	10	225.90	22.59			86.6
**Carbon cycling**						
Environment	6	763.41	127.23	1.05	0.295	-
Host Phyla	4	755.32	188.83	1.56	**0.001**	9.7
Residuals	17	2056.50	120.97			90.4
**Nitrogen cycling**						
Environment	6	174.59	29.10	0.94	0.733	-
Host Phyla	4	187.90	46.97	1.52	**0.002**	9.8
Residuals	15	464.23	30.95			90.2
**Mobility**						
Environment	6	35.14	5.86	0.85	0.780	-
Host Phyla	4	136.75	34.19	4.97	**0.001**	44.2
Residuals	16	110.18	6.89			55.8

*Significant *P*-values are boldface, indicates statistical significance with $P < 0.05$; *P*-values based on 999 permutations (lowest *P*-value possible 0.001); DF – degrees of freedom; SS – sum of squares; Pseudo-F – F value by permutation; ECV – estimated components of variation; Formula – Trait distance matrices ~ Environment + Host Phylum.
