# Supplementary material for: Plasmid-Encoded Traits Vary across Environments
Source: mBio. 2023 Jan 11;14(1):e03191-22. doi: 10.1128/mbio.03191-22 (PMC9973032; doi:10.1128/mbio.03191-22)
Supplement: FIG S6 [file mbio.03191-22-s0007.pdf]

A.

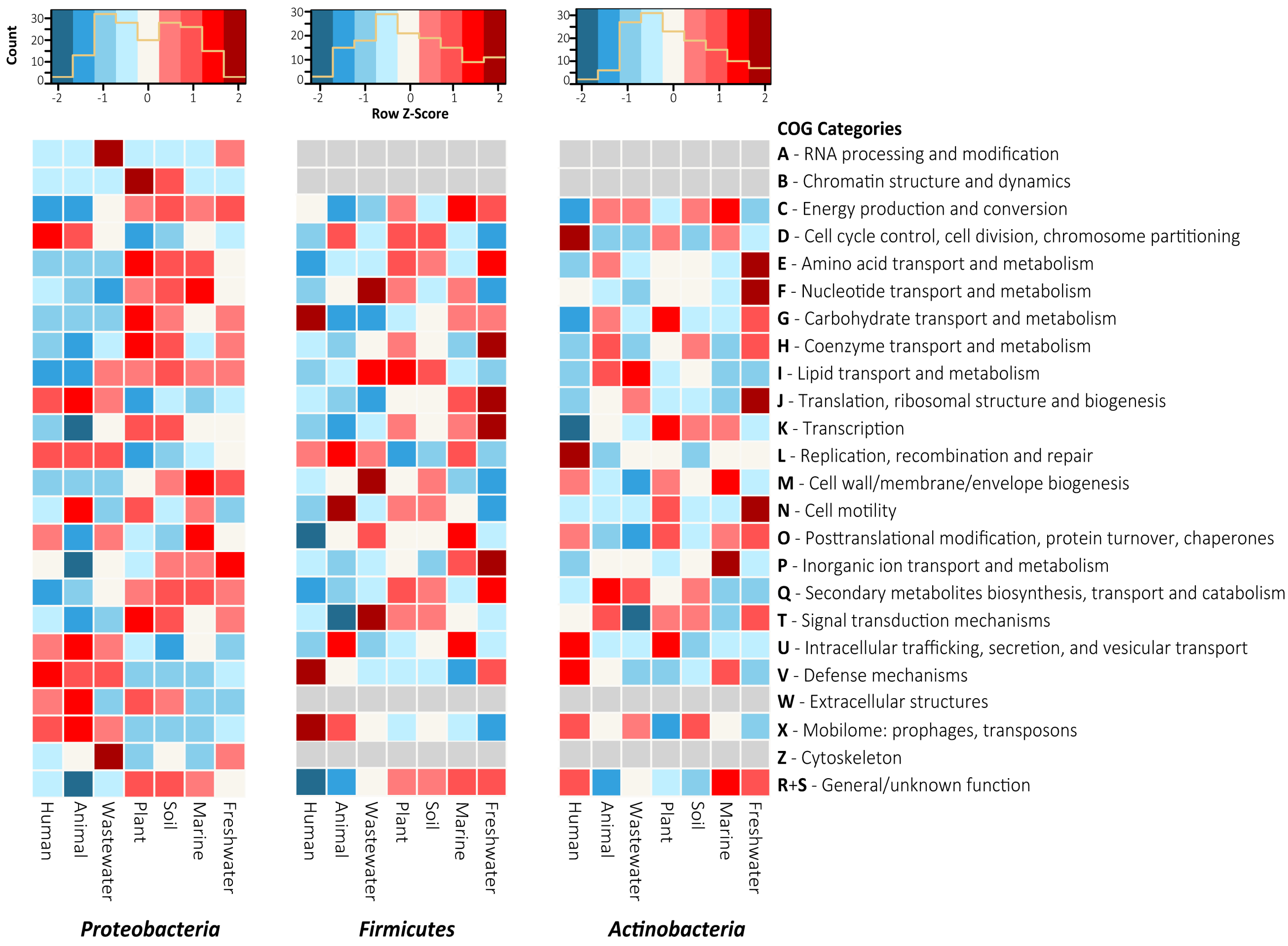
Count
Row Z-Score
COG Categories
A - RNA processing and modification
B - Chromatin structure and dynamics
C - Energy production and conversion
D - Cell cycle control, cell division, chromosome partitioning
E - Amino acid transport and metabolism
F - Nucleotide transport and metabolism
G - Carbohydrate transport and metabolism
H - Coenzyme transport and metabolism
I - Lipid transport and metabolism
J - Translation, ribosomal structure and biogenesis
K - Transcription
L - Replication, recombination and repair
M - Cell wall/membrane/envelope biogenesis
N - Cell motility
O - Posttranslational modification, protein turnover, chaperones
P - Inorganic ion transport and metabolism
Q - Secondary metabolites biosynthesis, transport and catabolism
T - Signal transduction mechanisms
U - Intracellular trafficking, secretion, and vesicular transport
V - Defense mechanisms
W - Extracellular structures
X - Mobilome: prophages, transposons
Z - Cytoskeleton
R+S - General/unknown function
Human
Animal
Wastewater
Plant
Soil
Marine
Freshwater
Proteobacteria
Firmicutes
Actinobacteria
