# Supplementary material for: Plasmid-Encoded Traits Vary across Environments
Source: mBio. 2023 Jan 11;14(1):e03191-22. doi: 10.1128/mbio.03191-22 (PMC9973032; doi:10.1128/mbio.03191-22)
Supplement: TABLE S2 [file mbio.03191-22-s0008.pdf]

Table S2. Summary of (A) COG categories associated with mobilizable plasmids (the presence of at least one MOB relaxase encoding genes), (B) resistance genes associated with mobilizable plasmids, (C) CAZymes by family associated with mobilizable plasmids, and (D) N-gene families associated with mobilizable plasmids.

COG category	Mobilizable plasmid	Nontransmissible plasmid	% COG on mobilizable plasmids
A	6	18	25
B	2	6	25
C	12634	17886	41.4
D	12758	10936	53.8
E	21187	31014	40.6
F	3052	5024	37.8
G	24115	31102	43.7
H	11021	15003	42.4
I	11726	19011	38.2
J	7553	8972	45.7
K	30081	36726	45
L	34257	23976	58.8
M	15628	21040	42.6
N	4311	8295	34.2
O	12555	11226	52.8
P	16794	21650	43.7
Q	4483	7820	36.4
T	15596	24192	39.2
U	18453	10947	62.8
V	20377	18773	52.1
W	367	623	37.1
X	54582	55283	49.7
Z	22	11	66.7
R+S	29990	38077	44.1

B.

Resistance class	Mobilizable	Nontransmissible	% Class associated with mobilizable plasmids
Aminoglycosides	61	53	53.5
Arsenic	43	57	43
Beta-lactams	1587	852	65.1
Biocide and metal	12	29	29.3
Cadmium	1	2	33.3
Cationic antimicrobial peptides	8	6	57.1
Copper	19	12	61.3
Drug and biocide	96	67	58.9
Fluoroquinolones	27	9	75
Fosfomycin	8	3	72.7
Glycopeptides	6	9	40
Iron	0	1	0
Lead	96	43	69.1
Lipopeptides	2	1	66.7
Mercury	659	302	68.6
Metal	256	136	65.3
Metronidazole	1	7	12.5
MLS	37	26	58.7
Multi-biocide	1	6	14.3
Multi-drug	5	5	50
Naphthoquinone	136	41	76.8
Nickel	9	11	45
Phenicol	6	9	40
Rifampin	2	11	15.4
Sulfonamides	25	8	75.8
Tellurium	1	0	100
Tetracyclines	8	9	47.1
Trimethoprim	7	7	50

C.

CAZyme ID	Mobilizable	Nontransmissible	% CAZymes associated with mobilizable plasmids
AA10	2	35	5.4
AA3	82	92	47.1
AA3_2	78	64	54.9
AA4	4	7	36.4
AA5_2	0	5	0
AA6	1	8	11.1
AA7	15	1	93.8
CBM0	1	0	100
CBM12	1	9	10
CBM13	24	55	30.4
CBM16	2	1	66.7
CBM2	5	88	5.4
CBM20	2	12	14.3
CBM21	0	1	0
CBM22	1	3	25
CBM23	0	2	0
CBM26	1	1	50
CBM27	0	1	0
CBM3	0	4	0
CBM32	14	108	11.5
CBM34	3	3	50
CBM35	5	1	83.3
CBM4	1	3	25
CBM40	0	1	0
CBM41	0	3	0
CBM42	0	2	0
CBM48	157	411	27.6
CBM5	49	227	17.8
CBM50	10	41	19.6
CBM51	2	6	25
CBM54	0	1	0
CBM56	0	1	0
CBM57	3	0	100
CBM6	1	2	33.3
CBM61	0	3	0
CBM63	0	1	0
CBM66	1	1	50
CBM67	3	1	75
CBM85	0	1	0

CAZyme ID	Mobilizable	Nontransmissible	% CAZymes associated with mobilizable plasmids
CBM9	0	2	0
CE0	10	6	62.5
CE1	56	29	65.9
CE11	3	3	50
CE12	0	4	0
CE14	29	15	65.9
CE15	7	1	87.5
CE16	0	3	0
CE2	0	1	0
CE3	0	3	0
CE4	133	201	39.8
CE5	17	18	48.6
CE6	0	2	0
CE7	5	8	38.5
CE8	7	86	7.5
CE9	10	23	30.3
GH0	183	344	34.7
GH1	72	112	39.1
GH10	2	18	10
GH102	0	7	0
GH103	49	102	32.5
GH104	4	6	40
GH105	43	42	50.6
GH106	8	4	66.7
GH108	11	19	36.7
GH109	158	42	79
GH110	0	1	0
GH112	1	0	100
GH113	1	1	50
GH114	1	2	33.3
GH115	1	2	33.3
GH117	2	0	100
GH12	6	15	28.6
GH123	0	1	0
GH125	1	2	33.3
GH126	1	0	100
GH127	29	33	46.8
GH128	0	1	0
GH13	5	6	45.5
GH13_10	0	1	0

CAZyme ID	Mobilizable	Nontransmissible	% CAZymes associated with mobilizable plasmids
GH13_11	0	2	0
GH13_14	0	1	0
GH13_16	49	124	28.3
GH13_18	20	8	71.4
GH13_19	0	2	0
GH13_20	2	1	66.7
GH13_21	1	0	100
GH13_23	7	21	25
GH13_26	38	145	20.8
GH13_27	0	1	0
GH13_28	1	0	100
GH13_29	1	5	16.7
GH13_3	1	7	12.5
GH13_30	0	2	0
GH13_31	30	16	65.2
GH13_32	0	1	0
GH13_33	1	0	100
GH13_36	0	1	0
GH13_4	0	2	0
GH13_5	1	3	25
GH13_8	1	0	100
GH130	11	5	68.8
GH133	1	3	25
GH135	2	80	2.4
GH136	4	1	80
GH137	1	1	50
GH139	1	0	100
GH140	6	0	100
GH141	0	1	0
GH142	1	0	100
GH143	1	0	100
GH144	11	3	78.6
GH145	2	0	100
GH146	0	1	0
GH148	24	0	100
GH15	47	55	46.1
GH151	0	5	0
GH154	51	22	69.9
GH159	0	1	0
GH16	80	42	65.6

CAZyme ID	Mobilizable	Nontransmissible	% CAZymes associated with mobilizable plasmids
GH17	0	3	0
GH18	2	25	7.4
GH19	27	15	64.3
GH2	176	102	63.3
GH20	50	51	49.5
GH23	2640	676	79.6
GH24	20	135	12.9
GH25	45	40	52.9
GH26	26	12	68.4
GH27	1	2	33.3
GH28	54	223	19.5
GH29	45	60	42.9
GH3	124	120	50.8
GH30	0	1	0
GH30_1	0	1	0
GH30_2	0	1	0
GH30_5	0	2	0
GH31	11	24	31.4
GH32	147	188	43.9
GH33	49	54	47.6
GH35	6	8	42.9
GH36	64	33	66
GH37	2	87	2.3
GH38	35	38	48
GH39	9	35	20.5
GH4	119	134	47.0
GH42	22	41	34.9
GH43	0	1	0
GH43_1	2	1	66.7
GH43_10	1	2	33.3
GH43_11	1	3	25
GH43_12	6	2	75
GH43_17	1	1	50
GH43_18	2	0	100
GH43_22	0	3	0
GH43_24	1	0	100
GH43_26	4	2	66.7
GH43_27	0	1	0
GH43_29	3	0	100
GH43_30	0	1	0

CAZyme ID	Mobilizable	Nontransmissible	% CAZymes associated with mobilizable plasmids
GH43_32	0	1	0
GH43_5	1	0	100
GH43_8	1	0	100
GH43_9	2	0	100
GH44	0	1	0
GH46	1	1	50
GH5	3	4	42.9
GH5_1	2	21	8.7
GH5_12	0	1	0
GH5_13	3	0	100
GH5_2	0	1	0
GH5_28	1	0	100
GH5_39	0	1	0
GH5_4	0	1	0
GH5_44	0	1	0
GH5_46	1	3	25
GH5_48	7	7	50
GH5_5	2	80	2.4
GH5_7	2	0	100
GH5_8	2	0	100
GH50	1	1	50
GH51	38	49	43.7
GH52	0	1	0
GH53	0	21	0
GH55	0	1	0
GH57	1	0	100
GH6	0	3	0
GH63	40	22	64.5
GH65	13	7	65
GH66	0	1	0
GH67	2	1	66.7
GH68	14	5	73.7
GH70	2	2	50
GH73	163	174	48.4
GH74	0	2	0
GH75	0	1	0
GH76	1	2	33.3
GH77	32	120	21.1
GH78	22	48	31.4
GH79	3	0	100

CAZyme ID	Mobilizable	Nontransmissible	% CAZymes associated with mobilizable plasmids
GH8	12	23	34.3
GH81	0	1	0
GH84	7	8	46.7
GH85	8	0	100
GH86	1	0	100
GH88	38	52	42.2
GH89	2	0	100
GH9	3	1	75
GH91	9	25	26.5
GH92	7	3	70
GH93	0	1	0
GH94	27	56	32.5
GH95	3	13	18.8
GH97	2	1	66.7
GT0	125	309	28.8
GT1	167	211	44.2
GT10	0	6	0
GT102	1	5	16.7
GT103	0	4	0
GT107	52	4	92.9
GT11	1	18	5.3
GT14	0	3	0
GT17	0	9	0
GT19	1	2	33.3
GT2	606	1014	37.4
GT20	31	187	14.2
GT21	8	4	66.7
GT23	17	2	89.5
GT25	6	15	28.6
GT26	19	58	24.7
GT28	2	12	14.3
GT3	1	1	50
GT30	1	3	25
GT32	2	88	2.2
GT35	25	36	41
GT38	1	0	100
GT39	0	1	0
GT4	626	883	41.5
GT41	19	109	14.8
GT42	1	0	100

CAZyme ID	Mobilizable	Nontransmissible	% CAZymes associated with mobilizable plasmids
GT44	30	121	19.0
GT45	1	0	100
GT5	39	96	28.9
GT51	94	82	53.4
GT53	3	0	100
GT56	0	1	0
GT6	1	0	100
GT60	0	3	0
GT7	0	6	0
GT70	0	1	0
GT73	15	2	88.2
GT75	0	1	0
GT8	11	13	45.8
GT81	1	2	33.3
GT83	24	116	17.1
GT87	2	1	66.7
GT89	0	2	0
GT9	5	126	3.8
GT99	0	2	0
PL0	3	92	3.2
PL1	9	6	60
PL1_2	25	0	100
PL1_4	0	1	0
PL1_6	2	1	66.7
PL10_1	2	1	66.7
PL11	0	1	0
PL12	1	3	25
PL15_1	0	3	0
PL22_1	0	3	0
PL22_2	1	0	100
PL26	0	1	0
PL3	0	3	0
PL3_1	26	1	96.3
PL3_2	9	2	81.8
PL33_1	31	21	59.6
PL4_1	0	2	0
PL5	0	4	0
PL7	0	1	0
PL8	2	4	33.3
PL9	0	5	0

CAZyme ID	Mobilizable	Nontransmissible	% CAZymes associated with mobilizable plasmids
PL9_1	30	3	90.9
PL9_2	1	20	4.8

D.

N-gene family ID	Mobilizable	Nontransmissible	% N-genes associated with mobilizable plasmids
amoC_B	0	1	0
ansB	7	17	29.2
asnB	1	1	50
gdh_K00260	4	3	57.1
gdh_K00261	11	9	55
gdh_K00262	120	143	45.6
gdh_K15371	5	6	45.5
glnA	597	770	43.7
glsA	16	13	55.2
gs_K00264	10	1	90.9
gs_K00265	16	28	36.4
gs_K00266	115	192	37.5
napA	9	18	33.3
napC	12	15	44.4
narB	23	10	69.7
narC	1	7	12.5
narG	1	6	14.3
narI	5	18	21.7
narJ	2	22	8.3
narY	0	1	0
narZ	5	6	45.5
nasA	7	28	20
nifD	55	4	93.2
nifH	8	1	88.9
nifK	95	17	84.8
nifW	3	0	100
nirA	18	53	25.4
nirB	8	7	53.3
nirD	109	97	52.9
nirK	55	106	34.2
nirS	110	162	40.4
nmo	87	135	39.2
norB	0	1	0
norC	6	14	30
nosZ	118	167	41.4
NR	121	67	64.4
nrfC	8	15	34.8
ureA	0	1	0
ureB	1	2	33.3
ureC	4	13	23.5
